# The structure of the first representative of Pfam family PF06475 reveals a new fold with possible involvement in glycolipid metabolism

**DOI:** 10.1107/S1744309109022684

**Published:** 2009-10-27

**Authors:** Constantina Bakolitsa, Abhinav Kumar, Daniel McMullan, S. Sri Krishna, Mitchell D. Miller, Dennis Carlton, Rafael Najmanovich, Polat Abdubek, Tamara Astakhova, Hsiu-Ju Chiu, Thomas Clayton, Marc C. Deller, Lian Duan, Ylva Elias, Julie Feuerhelm, Joanna C. Grant, Slawomir K. Grzechnik, Gye Won Han, Lukasz Jaroszewski, Kevin K. Jin, Heath E. Klock, Mark W. Knuth, Piotr Kozbial, David Marciano, Andrew T. Morse, Edward Nigoghossian, Linda Okach, Silvya Oommachen, Jessica Paulsen, Ron Reyes, Christopher L. Rife, Christina V. Trout, Henry van den Bedem, Dana Weekes, Aprilfawn White, Qingping Xu, Keith O. Hodgson, John Wooley, Marc-André Elsliger, Ashley M. Deacon, Adam Godzik, Scott A. Lesley, Ian A. Wilson

**Affiliations:** a Joint Center for Structural Genomics, http://www.jcsg.org, USA; b Program on Bioinformatics and Systems Biology, Burnham Institute for Medical Research, La Jolla, CA, USA; c Stanford Synchrotron Radiation Lightsource, SLAC National Accelerator Laboratory, Menlo Park, CA, USA; d Protein Sciences Department, Genomics Institute of the Novartis Research Foundation, San Diego, CA, USA; e Center for Research in Biological Systems, University of California, San Diego, La Jolla, CA, USA; f Department of Molecular Biology, The Scripps Research Institute, La Jolla, CA, USA; g Département de Biochimie, Université de Sherbrooke, Québec, Canada; h Photon Science, SLAC National Accelerator Laboratory, Menlo Park, CA, USA

**Keywords:** structural genomics, DUFs, glycolipids, osmotic stress, host–pathogen interactions

## Abstract

The crystal structure of PA1994 from *Pseudomonas aeruginosa*, a member of the Pfam PF06475 family classified as a domain of unknown function (DUF1089), reveals a novel fold comprising a 15-stranded β-sheet wrapped around a single α-helix that assembles into a tight dimeric arrangement. The remote structural similarity to lipoprotein localization factors, in addition to the presence of an acidic pocket that is conserved in DUF1089 homologs, phospholipid-binding and sugar-binding proteins, indicate a role for PA1994 and the DUF1089 family in glycolipid metabolism. Genome-context analysis lends further support to the involvement of this family of proteins in glycolipid metabolism and indicates possible activation of DUF1089 homologs under conditions of bacterial cell-wall stress or host–pathogen interactions.

## Abstract 2

PA1994, a Pfam PF06475 (DUF1089) family homolog from *P. aeruginosa*, reveals remote similarities to lipoprotein localization factors and a conserved putative glycolipid-binding site.

## 1. Introduction

In an effort to extend the structural coverage of proteins for which the biological function is unknown and cannot be deduced by homology (*i.e.* domains of unknown function; DUFs), targets were selected from Pfam protein family PF06745 (DUF1089). DUF1089 homologs are present in pathogenic actinobacteria, burkholderia, firmicutes and lactobacilli. Here, we report the crystal structure of PA1994, the first structural representative of this family, which was determined using the semi-automated high-throughput pipeline of the Joint Center for Structural Genomics (JCSG; http://www.jcsg.org; Lesley *et al.*, 2002 ▶) as part of the NIGMS Protein Structure Initiative (PSI; http://www.nigms.nih.gov/Initiatives/PSI/). The *PA1994* gene of *Pseudomonas aeruginosa*, an opportunistic human pathogen (Gomez & Prince, 2007 ▶), encodes a protein with a molecular weight of 21.6 kDa (residues 1–187) and a calculated isoelectric point of 4.9.

We show that global and local structural and chemical similarities to lipid-binding proteins suggest the involvement of PA1994 with the bacterial membrane, while genome-context analysis supports a role for the DUF1089 family in glycolipid metabolism that is likely to be triggered under conditions of osmotic stress or host–pathogen interactions. These structural insights should help to guide future functional studies.

## 2. Materials and methods

### 2.1. Protein production and crystallization

Clones were generated using the Polymerase Incomplete Primer Extension (PIPE) cloning method (Klock *et al.*, 2008 ▶). The gene encoding PA1994 (GenBank NP_250684; gi:15597190; Swiss-Prot Q912B5) was amplified by polymerase chain reaction (PCR) from *P. aeruginosa* PA01-LAC genomic DNA using *PfuTurbo* DNA polymerase (Stratagene) and I-PIPE (Insert) primers (forward primer, 5′-ctgtacttccagggcATGAGTCGCGACCGTCTGTACACCT­GGG-3′; reverse primer, 5′-aattaagtcgcgttaGAGACGCTGGAAG­AGACCCGGGTAATCG-3′; target sequence in upper case) that included sequences for the predicted 5′ and 3′ ends. The expression vector pSpeedET, which encodes an amino-terminal tobacco etch virus (TEV) protease-cleavable expression and purification tag (MGSDKIHHHHHHENLYFQ/G), was PCR-amplified with V-PIPE (Vector) primers (forward primer, 5′-taacgcgacttaattaactcgtttaaacgg­tctccagc-3′; reverse primer, 5′-gccctggaagtacaggttttcgtgatgatgatgatg­atg-3′). V-PIPE and I-PIPE PCR products were mixed to anneal the amplified DNA fragments. *Escherichia coli* GeneHogs (Invitrogen) competent cells were transformed with the V-PIPE/I-PIPE mixture and dispensed onto selective LB–agar plates. The cloning junctions were confirmed by DNA sequencing. Expression was performed in selenomethionine-containing medium with suppression of normal methionine synthesis. At the end of fermentation, lysozyme was added to the culture to a final concentration of 250 µg ml^−1^ and the cells were harvested and frozen. After one freeze–thaw cycle, the cells were sonicated in lysis buffer [50 m*M* HEPES pH 8.0, 50 m*M* NaCl, 10 m*M* imidazole, 1 m*M* tris(2-carboxyethyl)phosphine–HCl (TCEP)] and the lysate was clarified by centrifugation at 32 500*g* for 30 min. The soluble fraction was passed over nickel-chelating resin (GE Healthcare) pre-equilibrated with lysis buffer, the resin was washed with wash buffer [50 m*M* HEPES pH 8.0, 300 m*M* NaCl, 40 m*M* imidazole, 10%(*v*/*v*) glycerol, 1 m*M* TCEP] and the protein was eluted with elution buffer [20 m*M* HEPES pH 8.0, 300 m*M* imidazole, 10%(*v*/*v*) glycerol, 1 m*M* TCEP]. The eluate was buffer-exchanged with HEPES crystallization buffer (20 m*M* HEPES pH 8.0, 200 m*M* NaCl, 40 m*M* imidazole, 1 m*M* TCEP) using a PD-10 column (GE Healthcare) and incubated with 1 mg TEV protease per 15 mg eluted protein. The protease-treated eluate was passed over nickel-chelating resin (GE Healthcare) pre-equilibrated with HEPES crystallization buffer and the resin was washed with the same buffer. The flowthrough and wash fractions were combined and concentrated to 11.2 mg ml^−1^ by centrifugal ultrafiltration (Millipore) for crystallization trials. PA1994 was crystallized using the nanodroplet vapor-diffusion method (Santarsiero *et al.*, 2002 ▶) with standard JCSG crystallization protocols (Lesley *et al.*, 2002 ▶). Sitting drops composed of 200 nl protein solution mixed with 200 nl crystallization solution were equilibrated against a 50 µl reservoir at 277 K for 40 d prior to harvesting. Initial screening for diffraction was carried out using the Stanford Automated Mounting system (SAM; http://smb.slac.stanford.edu/facilities/hardware/SAM/UserInfo; Cohen *et al.*, 2002 ▶) at the Stanford Synchrotron Radiation Lightsource (SSRL; Menlo Park, California, USA). The crystallization reagent that produced the PA1994 crystal used for the structure solution con­tained 5%(*v*/*v*) 2-­methyl-2,4-pentanediol (MPD; racemic mixture), 10%(*w*/*v*) PEG 6000 and 0.1 *M* HEPES pH 7.5. Ethylene glycol was added to the crystal as a cryoprotectant to a final concentration of 15%(*v*/*v*). A rod-shaped crystal with approximate dimensions of 200 × 20 × 20 µm was mounted in a nylon loop. The diffraction data were indexed in the monoclinic space group *C*2 (Table 1 ▶). The molecular weight and oligomeric state of PA1994 were determined using a 0.8 × 30 cm Shodex Protein KW-­803 column (Thomson Instruments) pre-calibrated with gel-filtration standards (Bio-Rad).

### 2.2. Data collection, structure solution and refinement

Multiple-wavelength anomalous diffraction (MAD) data were collected at SSRL on beamline BL11-1 at wavelengths corresponding to the inflection (λ_1_), peak (λ_2_) and high-energy remote (λ_3_) of a selenium MAD experiment. The data sets were collected at 100 K with an ADSC Q315 CCD detector using the *Blu-Ice* data-collection environment (McPhillips *et al.*, 2002 ▶). The MAD data were integrated and reduced using *XDS* and then scaled with the program *XSCALE* (Kabsch, 1993 ▶). Phasing was performed with *SHELX* (Sheldrick, 2008 ▶) and *AutoSHARP* (Bricogne *et al.*, 2003 ▶), which resulted in a mean figure of merit of 0.15 with four selenium positions. Two were high occupancy, corresponding to the main selenium positions at residues *A*143 and *B*143, whereas the others were low occupancy (20% relative to the primary site), corresponding to an alternate conformation of residue 143 in each monomer (<4.7 Å from the primary site). It should be noted that the presence of only one ordered SeMet site (two conformations) per 188 residues in the protein chain sufficed for successful phasing and model building. Automated model building was performed with *ARP*/*wARP* (Cohen *et al.*, 2004 ▶) and model completion and refinement were performed with *Coot* (Emsley & Cowtan, 2004 ▶) and *REFMAC* 5.2 (Winn *et al.*, 2003 ▶). Refinement included phase restraints from *AutoSHARP* and TLS refinement with two TLS groups per chain as suggested by the *TLSMD* server (Painter & Merritt, 2006 ▶). Data reduction and refinement statistics are summarized in Table 1 ▶.

### 2.3. Validation and deposition

**Figure 1:**
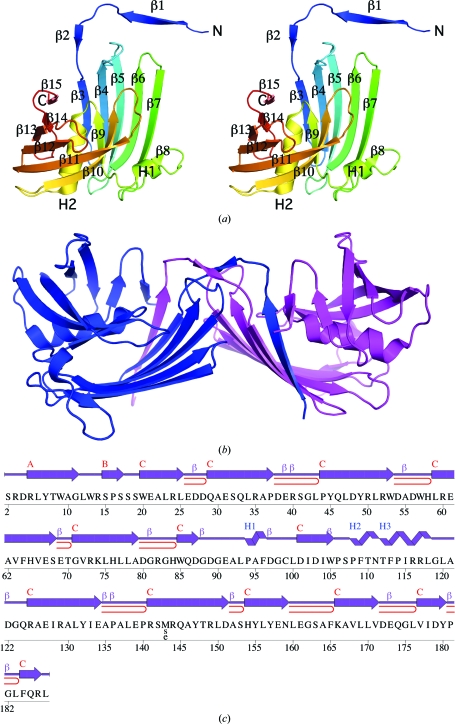
Crystal structure of PA1994 from *P. aeruginosa*. (*a*) Stereo ribbon diagram of the PA1994 monomer color coded from the N-terminus (blue) to the C-terminus (red). Helices (H1–H2) and β-strands (β1–β15) are indicated. (*b*) Ribbon representation of the PA1994 dimer showing domain swapping of the N-terminal β-strands. Monomers are depicted in blue and magenta. (*c*) Diagram showing the secondary-structure elements of PA1994 superimposed on its primary sequence. The labeling of secondary-structure elements is in accord with *PDBsum* (http://www.ebi.ac.uk/pdbsum), where α-helices are sequentially labeled (H1, H2, H3 *etc*.), β-strands are labeled (A, B, C *etc*.) according to the β-sheets to which they are assigned, β-turns and γ-turns are designated by Greek letters (β, γ) and β-hairpins are designated by red loops. For PA1994, the α-helix (H2), 3_10_-helix (H1), β-strands in β-sheets (A–C), β-turns (β) and β-hairpins are indicated.

Analysis of the stereochemical quality of the model was accomplished using *AutoDepInputTool* (Yang *et al.*, 2004 ▶), *MolProbity* (Davis *et al.*, 2004 ▶), *SFCHECK* 4.0 (Collaborative Computational Project, Number 4, 1994 ▶) and *WHATIF* 5.0 (Vriend, 1990 ▶). Protein quaternary-structure analysis was performed using the *PISA* server (Krissinel & Henrick, 2007 ▶). Fig. 1 ▶(*c*) was adapted from an analysis using *PDBsum* (Laskowski *et al.*, 2005 ▶) and all other figures were prepared with *PyMOL* (DeLano Scientific). Atomic coordinates and experimental structure factors for PA1994 at 1.80 Å resolution have been deposited in the PDB under accession code 2h1t.

## 3. Results and discussion

### 3.1. Overall structure

The crystal structure of PA1994 (Fig. 1 ▶ *a*) was determined to 1.80 Å resolution using the multiple-wavelength anomalous dispersion (MAD) method. Refinement statistics are summarized in Table 1 ▶. The final model includes 370 residues (residues 2–187 of chain *A* and residues 4–187 of chain *B*), nine ethylene glycol molecules, two MPD molecules and 367 water molecules in the asymmetric unit. No electron density was observed for the N-terminal glycine (0) remaining after cleavage of the expression and purification tag, for the terminal selenomethionine (residue 1) of chains *A* and *B* or for Ser2 and Arg3 in chain *B*. The side chains of Arg5 and Glu91 in chain *B* were omitted owing to weak electron density. The Matthews coefficient (*V*_M_; Matthews, 1968 ▶) was 2.5 Å^3^ Da^−1^ and the estimated solvent content was 50.1%. A Ramachandran plot produced by *MolProbity* (Davis *et al.*, 2004 ▶) showed that 99.2% of the residues are in favored regions. The two outliers, Pro106 in chains *A* and *B*, are actually found in a *cis* conformation in both chains and have clear electron density.

*SCOP* (release 1.75) classifies PA1994 as a single-domain protein with a novel fold termed a spiral β-roll (http://scop.mrc-lmb.cam.ac.uk/scop/data/scop.b.c.bdb.b.b.b.html), with a 15-stranded β-sheet wrapped around a central helix (Fig. 1 ▶). The N-terminal half of the sheet is formed by strands β3–β7 supplemented by a β1-strand exchange from the other monomer in the asymmetric unit (Fig. 1 ▶ *b*) that hydrogen bonds extensively to the β3 and the shorter β15 strands (Figs. 1 ▶ *a* and 1 ▶ *b*). This swapping additionally involves strand β2 and results in a large buried dimerization interface of ∼3000 Å^2^ per monomer. A short β-­strand (β8) and 3_10_-helix H1 separate the first half of the β-sheet from the more tightly curved C-terminal region (strands β10–β15). Helix H2 and strand β9 are sandwiched between the two halves of the β-sheet in the center of the molecule.

PA1994 can be viewed as consisting of two subdomains: the first half of the β-sheet (β1′, β3–β8) and helix H1 (residues 1–98) compose the first domain, which packs against the other subdomain consisting of the second half of the β-sheet (β9–β15) and helix H2 (residues 99–187). Both subdomains are present in DUF1089-family members and a sequence analysis of the family indicates a high degree of conservation in the residues that are implicated in stabilizing both regions of the molecule. Stacking interactions, both intermolecular (Trp9–Pro108′) and intramolecular (Trp57–Phe113), show strict or high conservation. Additionally, conserved stacking interactions are observed in residue pairs involving the H2 helix and both the N-­terminal (Trp57–Phe113) and the C-terminal (Pro114–Tyr147) halves of the β-­sheet, as well as the conserved binding-pocket residues (Trp9–Pro108′, Trp57–Phe113 and Pro106/Pro108–Phe184; see below).

A search with *FATCAT* (Ye & Godzik, 2004 ▶) identified that the highest structural similarity is with outer membrane proteins (SH3-like barrel fold), NTF2-like proteins (cystatin-like fold) and fatty acid-binding proteins (lipocalin fold). *DALI* (Holm & Sander, 1995 ▶) showed significant hits with a number of different folds, including β-­galactosidase (immunoglobulin-like β-sandwich fold), iron-transport proteins (transmembrane β-barrel fold), lipovitellin (lipo­vitellin–phosvitin complex/β-sheet shell regions fold), tail-associated lysozyme (phage-tail protein fold) and lipoprotein localization factors (prokaryotic lipoprotein localization factor fold). A search using secondary-structure matching (*SSM*; Krissinel & Henrick, 2004 ▶) identified the lipoprotein localization factor LolA (PDB code 1iwl) as the top hit (*Z* score 2.5, *P* score 0.0), although the *P* score indicates a statistically insignificant match.

**Figure 2:**
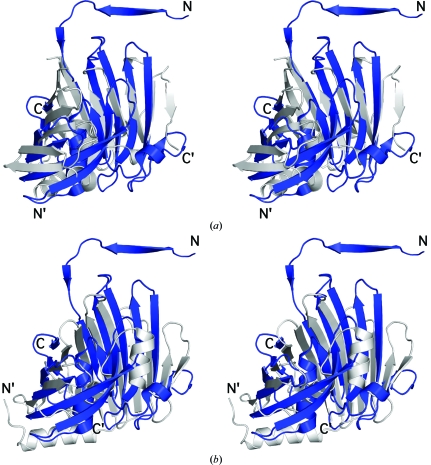
PA1994 exhibits structural similarity to the lipoprotein chaperones LolA and LolB. (*a*) Stereoview of the structural superposition of PA1994 (PDB code 2h1t, residues 2–187, blue) and LolA (PDB code 1iwl, residues 1–182, gray). (*b*) Stereoview of the structural superposition of PA1994 (PDB code 2h1t, residues 2–187, blue) and LolB (PDB code 1iwn, residues 10–186, gray).

Although PA1994 appears to constitute a new fold, we decided to investigate subfold similarities in an attempt to identify shared structural features that could provide insight into the origin and function of PA1994. The highest structural similarity identified by visual inspection was with lipoprotein localization factors A and B (LolA and LolB) from *E. coli*, which are highly conserved bacterial proteins that are implicated in lipoprotein sorting and membrane localization (Takeda *et al.*, 2003 ▶). Superimposition of PA1994 onto LolA, with an r.m.s.d. of 3.1 Å, reveals that these proteins share the same fold and topology over the 11 β-strands and the central helix, although the sequence identity over 104 aligned residues is not significant at only 5% (Fig. 2 ▶ *a*). Differences within the barrel include PA1994 strands β9–β10, which are absent in both lipoprotein local­ization factors, strand β8 (absent in LolA) and the orientation of the central helix in LolB (Figs. 2 ▶ *a* and 2 ▶ *b*). Outside the barrel, the main differences involve an additional N-terminal helix in LolA located at the bottom of the β-barrel and the LolA C-terminal 3_10_-helix and β-­strand (Figs. 2 ▶ *a* and 2 ▶ *b*). Both of these C-terminal structural elements, which are absent in PA1994, are involved in the specific membrane localization of lipoproteins by LolA (Okuda *et al.*, 2008 ▶). No strand-swapping is observed in either LolA or LolB, although the N-terminal β-strand is present in both cases and overlaps with the swapped strand from the PA1994 dimer.

### 3.2. Analysis of a conserved cavity

**Figure 3:**
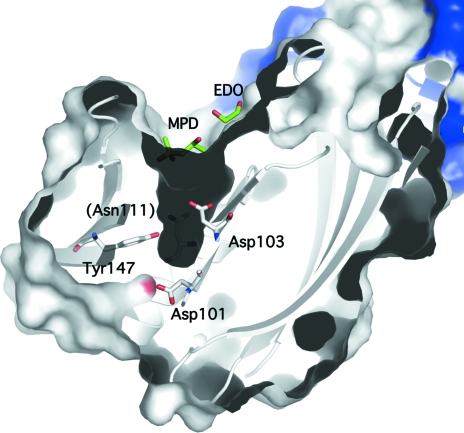
An acidic pocket conserved in the DUF1089 family suggests a ligand-binding site. The PA1994 monomers, colored white and blue, are shown as a ribbon diagram and as a surface representation. Invariant residues (Asp101, Asp103 and Tyr147) are indicated, with the conserved Asn111 located behind the pocket labeled in parentheses. The ethylene glycol (EDO) and MPD molecules that line the entrance to the acidic pocket in the crystal are shown in green.

An analysis of PA1994 using the *CastP* server (Binkowski *et al.*, 2003 ▶) revealed a deep pocket (15 × 6 × 7 Å) enclosed mainly by helix H2 and strand β7, with additional contributions made by strands β10–β12 and the loop between strands β14 and β15. This pocket is lined with conserved hydrophilic residues (Ser107, Thr110, Asn111, Thr112 and Gln145) and contains the hydroxyl group of the invariant Tyr147 in addition to an acidic pocket formed by two invariant aspartates (Asp101 and Asp103; Fig. 3 ▶). The pocket is in a similar location to the cavity in LolA that has been shown to bind lipids (Watanabe *et al.*, 2006 ▶). However, the binding pocket is hydrophobic in LolA, whereas the PA1994 pocket is acidic, suggesting a more hydrophilic ligand. The entrance to the pocket in PA1994 forms a long and narrow groove (20 × 7 Å) composed of strictly or highly conserved hydrophobic residues (Ile102, Pro106, Pro108, Phe165, Leu170 and Ile178) and also involves the dimerization interface (Trp13), suggesting a hydrophobic component of the ligand and the likely requirement of dimerization for binding. Analytical size-exclusion chromatography in combination with static light scattering indicates that PA1994 is a dimer in solution. Two crystallization-reagent molecules (ethylene glycol and MPD) line both the groove and the pocket, indicating that both regions could be implicated in ligand binding (Fig. 3 ▶ *b*). Both LolA and PA1994 contain a *cis*-proline (Pro89 in LolA and Pro106 in PA1994) at the N-terminal end of the central helix. Because of the relatively low energy barrier between *trans* and *cis* conformations, *cis*-prolines are often involved in function and have been implicated in both protein stabilization (Truckses *et al.*, 1996 ▶) and catalysis (Charbonnier *et al.*, 1999 ▶), suggesting that this residue might serve a similar purpose in LolA and PA1994.

A search against a database of nonredundant cognate binding sites using *IsoCleft* (Najmanovich *et al.*, 2008 ▶), a graph-matching algorithm that searches for both geometrical and chemical composition similarities, identified shared features between the PA1994 pocket and the binding sites of proteins implicated in bacterial cell-wall biosynthesis, with alanine racemase from *P. aeruginosa* (PDB code 1rcq; 21 atoms in common, Tanimoto similarity score 0.39, *Z* score 4.26, *P* value 7.54 × 10^−3^; LeMagueres *et al.*, 2003 ▶) and hyaluronate lyase from *Streptococcus pneumoniae* (PDB code 1loh; 21 atoms in common, Tanimoto similarity score 0.38, *Z* score 4.01, *P* value 1.03 × 10^−2^; Jedrzejas *et al.*, 2002 ▶) as the top hits. Additional similarities include the binding of sugars, with galactose mutarotase (PDB code 1so0; 25 atoms in common, Tanimoto similarity score 0.38, *Z* score 4.08, *P* value 9.44 × 10^−3^; Thoden *et al.*, 2004 ▶) and *meso*-2,3-butane­diol dehydrogenase (PDB code 1geg; 20 atoms in common, Tanimoto similarity score 0.36, *Z* score 3.82, *P* value 1.32× 10^−2^; Otagiri *et al.*, 2001 ▶) as the closest matches, in addition to an inorganic pyrophos­phatase (PDB code 1wpm; 25 atoms in common, Tanimoto similarity score 0.37, *Z* score 3.89, *P* value 1.21 × 10^−2^; Fabrichniy *et al.*, 2004 ▶). *IsoCleft* also identified similarities between the hydrophobic groove along the PA1994 pocket entrance and dimerization interface and the lipid-binding site in *Candida rugosa* lipase (PDB code 1lpn; 31 atoms in common, Tanimoto similarity score 0.20, *Z* score 3.98, *P* value 1.08 × 10^−2^; Grochulski, Bouthillier *et al.*, 1994 ▶).

Taken together, these structural and chemical similarities support a role for PA1996 and the DUF1089 family in glycolipid binding. The extensive dimerization interface observed in the structure, in addition to the SEC/SLS data, suggest that a dimer is likely to be the bio­logically relevant oligomeric state of PA1994. The swapped β-strands appear to participate in stabilizing the conserved cavity. Substrate binding might induce large-scale conformational changes, as is the case for the lipid-binding proteins that share structural similarities with PA1994 (Marland *et al.*, 2006 ▶; Oguchi *et al.*, 2008 ▶; Grochulski, Li *et al.*, 1994 ▶).

### 3.3. Genome-context analysis

Glycophospholipids, which are implicated in the synthesis of complex cell-wall structures that enable some pathogens to modulate the response by the host immune system, have been suggested to bind to similar-sized acidic pockets as that observed in PA1994 (Marland *et al.*, 2006 ▶). Glycolipids serve as key immunomodulatory molecules in host–pathogen interactions (Nigou *et al.*, 2008 ▶) and lipases have been known to act as virulence factors (Smoot, 1997 ▶). In addition to their role in pathogenicity, bacterial cell-wall glycolipids are modified in response to variations in temperature, pH and other environmental stressors (Mykytczuk *et al.*, 2007 ▶), with changes affecting both the lipid and sugar composition of the membrane (Bengoechea *et al.*, 2002 ▶; Tymczyszyn *et al.*, 2005 ▶).

The genome context (http://string.embl.de) of DUF1089-family members additionally supports a role in glycolipid biosynthesis which is likely to be induced under conditions of cell-wall stress or host–pathogen interactions. PA1994 is predicted with a high degree of confidence to be in functional association with a peptidyl prolyl *cis*–*trans* isomerase (PA1996), an enzyme that functions as a chaperone and is up-regulated under conditions of cell-wall stress (Muthaiyan *et al.*, 2008 ▶). The prolyl *cis*–*trans* isomerase could also assist in the folding of PA1994, as Pro106 appears to be involved in stabilization of both the hydrophobic core and the acidic pocket. Similarly, R02764, a DUF1089 homologue from *Sinorhizobium meliloti*, is predicted to be functionally linked to a glyceraldehyde 3-phosphate dehydrogenase [R02763, normally a cytosolic enzyme involved in energy metabolism that shows pH-dependent association with bacterial cell walls (Antikainen *et al.*, 2007 ▶), where it becomes involved in host–pathogen interactions (Schaumburg *et al.*, 2004 ▶)], a transketolase (R02762, an enzyme implicated in lipopolysaccharide metabolism; Eidels & Osborn, 1971 ▶) and a taurine-uptake ABC transporter (RB0965; taurine is a constituent of the bacterial cell wall that has been implicated in membrane stabilization and recovery from osmotic shock; Yancey, 2005 ▶). MT3862, a DUF1089 homologue from *Mycobacterium tuberculosis*, is also predicted with high confidence to be in functional association with two osmoprotectant proteins (MT3863 and MT3864) implicated in glycine betaine-dependent transport. In addition to its role in maintaining membrane fluidity, glycine betaine acts as a chemical chaperone (Diamant *et al.*, 2001 ▶), stabilizing proteins under conditions of environmental stress.

Availability of more DUF1089-member sequences and structures might shed light on the evolutionary history of this intriguing protein family. The information presented here, in combination with further biochemical and biophysical studies, should yield valuable insights into the functional role of PA1994. Models of PA1994 homologs can be accessed at http://www1.jcsg.org/cgi-bin/models/get_mor.pl?key=2hltA.

Additional information about PA1994 is available from TOPSAN (Krishna *et al.*, 2010 ▶) http://www.topsan.org/explore?PDBid=2h1t.

## 4. Conclusions

The first structural representative of the DUF1089 family reveals a novel fold. Remote global and local similarities to lipid-binding and glycan-binding proteins along with genome-context analysis support a role for PA1994 in glycolipid metabolism that is likely to be induced under conditions of cell-wall stress or host–pathogen interactions.

## Supplementary Material

PDB reference: PA1994 from *P. aeruginosa*, 2h1t, r2h1tsf

## Figures and Tables

**Table 1:** Summary of crystal parameters, data collection and refinement statistics for PA1994 (PDB code 2h1t) Values in parentheses are for the highest resolution shell.

	λ_1_ MADSe	λ_2_ MADSe	λ_3_ MADSe
Space group	*C*2		
Unit-cell parameters (Å, °)	*a* = 130.03, *b* = 41.90, *c* = 78.65, β = 91.2		
Data collection			
Wavelength (Å)	0.9793	0.9789	0.9116
Resolution range (Å)	28.3–1.80 (1.85–1.80)	28.3–1.91 (1.96–1.91)	28.3–1.80 (1.85–1.80)
No. of observations	136388	121791	146173
No. of unique reflections	38719	33103	39473
Completeness (%)	98.0 (83.9)	99.6 (97.2)	99.7 (98.3)
Mean *I*/σ(*I*)	9.9 (1.9)	10.6 (3.4)	10.3 (2.6)
*R*_merge_ on *I*† (%)	9.9 (51.4)	10.5 (35.1)	9.9 (51.7)
Model and refinement statistics			
Resolution range (Å)	28.3–1.80		
No. of reflections (total)	35699‡		
No. of reflections (test)	1772		
Completeness (%)	90.2		
Data set used in refinement	λ_1_ MADSe		
Cutoff criterion	|*F*| > 0		
*R*_cryst_§	0.170		
*R*_free_¶	0.213		
Stereochemical parameters			
Restraints (r.m.s.d. observed)			
Bond angles (°)	1.58		
Bond lengths (Å)	0.015		
Average isotropic *B* value (Å^2^)	20.5††		
ESU‡‡ based on *R*_free_ (Å)	0.13		
Protein residues/atoms	370/3051		
Waters/other solvent molecules	367/11		

† *R*_merge_ = 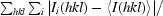 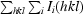.

‡ Owing to ice rings, a total of 3016 reflections were omitted from the resolution ranges 1.91–1.93, 2.02–2.04 and 2.23–­2.27 Å. Typically, a few reflections were also excluded owing to negative intensities and rounding errors in the resolution limits and unit-cell parameters.

§ *R*_cryst_ = 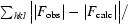 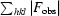, where *F*_calc_ and *F*_obs_ are the calculated and observed structure-factor amplitudes, respectively

¶ *R*_free_ is the same as *R*_cryst_ but for 5.0% of the total reflections chosen at random and omitted from refinement.

†† This represents the total *B* including both the TLS and residual *B* components.

‡‡ Estimated overall coordinate error (Collaborative Computational Project, Number 4, 1994 ▶; Cruickshank, 1999 ▶).
